# High frequency of brain metastases after adjuvant therapy for high‐risk melanoma

**DOI:** 10.1002/cam4.1223

**Published:** 2017-10-10

**Authors:** Wolfram E. Samlowski, James Moon, Merle Witter, Michael B. Atkins, John M. Kirkwood, Megan Othus, Antoni Ribas, Vernon K. Sondak, Lawrence E. Flaherty

**Affiliations:** ^1^ Comprehensive Cancer Centers of Nevada/Southern Nevada CCOP Las Vegas Nevada; ^2^ SWOG Statistical Center Seattle Washington; ^3^ Georgetown‐Lombardi Comprehensive Cancer Center Washington DC; ^4^ University of Pittsburgh Medical Center Pittsburgh Pennsylvania; ^5^ UCLA Medical Center Los Angeles California; ^6^ H Lee Moffitt Cancer Center Tampa Florida; ^7^ Wayne State University/Karmanos Cancer Institute Detroit Michigan

**Keywords:** Biochemotherapy, brain metastases, interferon, lymph node metastases, melanoma, ulceration

## Abstract

The incidence of CNS progression in patients with high‐risk regional melanoma (stages IIIAN2a‐IIIC) is not well characterized. Data from the S0008 trial provided an opportunity to examine the role of CNS progression in treatment failure and survival. All patients were surgically staged. Following wide excision and full regional lymphadenectomy, patients were randomized to receive adjuvant biochemotherapy (BCT) or high‐dose interferon alfa‐2B (HDI). CNS progression was retrospectively identified from data forms. Survival was measured from date of CNS progression. A total of 402 eligible patients were included in the analysis (BCT: 199, HDI: 203). Median follow‐up (if alive) was over 7 years (range: 1 month to 11 years). The site of initial progression was identifiable in 80% of relapsing patients. CNS progression was a component of systemic melanoma relapse in 59/402 patients (15% overall). In 34/402 patients (9%) CNS progression represented the initial site of treatment failure. CNS progression was a component of initial progression in 27% of all patients whose melanoma relapsed (59/221). The risk of CNS progression was highest within 3 years of randomization. The difference in CNS progression rates between treatment arms was not significant (BCT = 25, HDI = 34, *P *=* *0.24). Lymph node macrometastases strongly associated with CNS progression (*P *=* *0.001), while ulceration and head and neck primaries were not significant predictors. This retrospective analysis of the S0008 trial identified a high brain metastasis rate (15%) in regionally advanced melanoma patients. Further studies are needed to establish whether screening plus earlier treatment would improve survival following CNS progression.

## Introduction

Melanoma has the highest incidence proportion percentage of brain involvement across all solid tumors 1, and accounts for 6–11% of all patients who develop brain metastases 2. With the advent of modern imaging technologies (e.g., CT and MRI scans), brain metastases are detected in 15–30% of patients at initial diagnosis of metastatic disease 3, 4.

In patients with *metastatic* melanoma without brain metastases at initial presentation, a high risk of CNS progression has been reported. A recent phase III trial compared first‐line dacarbazine or temozolomide treatment for stage IV melanoma and excluded all patients with preexisting CNS metastases 5. By 1 year, CNS progression was detected in 20.6% and 31.1% temozolomide‐ or dacarbazine‐treated patients, respectively. The frequency of CNS progression increased to 32% and 45% by 3 years. The modest difference in CNS metastases between treatments was not significant (*P *=* *0.22). The probability of CNS progression increases with disease duration. As many as 75–90% of stage IV melanoma patients are found to have brain metastases at autopsy 6. Thus, brain metastases represent an extremely frequent complication of metastatic melanoma.

The development of CNS progression in early stage (Stage I and II) primary cutaneous melanoma following surgical resection appears to be relatively low 7. Ulceration and head and neck primaries have been identified as potential risk factors for CNS metastases in patients with early stage primary melanoma 7. There is little data about the incidence of CNS metastases in regionally advanced disease (stages IIIAN2a‐IIIC). We hypothesized that a significant percentage of these patients would experience CNS progression.

In order to assess the incidence of brain metastases in patients with high‐risk regional melanoma, we performed a retrospective review of study records from a large prospective randomized multi‐institutional clinical trial 8. Patients (*n *=* *402) with stages IIIAN2a‐IIIC melanoma enrolled on the S0008 trial were all uniformly staged and treated according to protocol guidelines. At a median follow‐up of 7.2 years, biochemotherapy (BCT) improved relapse‐free survival (RFS; hazard ratio [HR], 0.75; 95% CI, 0.58–0.97; *P *=* *0.015), with a median 4.0 years (95% CI, 1.9 years to not reached [NR]) versus 1.9 years for HDI (95% CI, 1.2–2.8 years) and a 5‐year RFS of 48% versus 39%. Median OS was not different (HR, 0.98; 95% CI, 0.74–1.31; *P *=* *0.55), with a median OS of 9.9 years (95% CI, 4.62 years to NR) for BCT versus 6.7 years (95% CI, 4.5 years to NR) for HDI and a 5‐year OS of 56% for both arms. The data forms included a space to identify the initial site of progression. This dataset allowed the incidence of brain metastases to be analyzed.

## Patients and Methods

Eligibility criteria for the S0008 trial have been previously published 8. In brief, patients were required to have skin melanoma or an unknown primary. Patients had to fulfill one of the following criteria related to their tumor extent: (1) an ulcerated primary melanoma with one or more regional lymph node micrometastases; (2) a nonulcerated melanoma with two or more regional lymph node micrometastases; (3) one or more clinically apparent macroscopic regional lymph node metastasis (including a single matted nodal mass); (4) initial satellite/in‐transit metastasis with or without regional lymph node involvement; (5) histologically confirmed: regional nodal recurrence; (6) recurrent disease in the lymph node basin following a previous lymphadenectomy; or (7) recurrent melanoma presenting with satellite/in‐transit metastases. Patients with resected or active distant metastases were not eligible for enrollment. This incorporated stage stages IIIAN2a‐IIIC disease (Table 1).

**Table 1:** Nodal classification for cutaneous melanoma—AJCC 6th Edition, 2002 [18]

*N* classification^c^	Description	Type of nodal involvement	S0008 patients
N1	1 lymph node involved	a: micrometastasis^a^ b: macrometastasis^b^	152 total N1 or N2
N2	2–3 lymph nodes involved	a: micrometastasis^a^ b: macrometastasis^b^ c: in‐transit met(s)/satellite(s) without involved nodes	
N3	4 or more lymph nodes involved, matted lymph nodes or combinations of in‐transit met(s)/satellite(s) with involved lymph node(s)		250

	T	*N*	M	5‐year survival	10‐year survival
Stage groupings (2002)					
IIIA					
T1‐4a		N1a	M0	70%	60%
T1‐4a		N2a	M0		
IIIB					
T1‐4b		N2a	M0	52%	40%
T1‐4a		N1b	M0		
T1‐4a		N2b	M0		
T1‐4a		N2c	M0		
IIIC					
T1‐4b		N2c	M0	25%	22%
Any T		N3	M0		

a Micrometastases are diagnosed after elective or sentinel lymphadenectomy.

b Macrometastases are defined as clinically detectable lymph node metastases confirmed by therapeutic lymphadenectomy or when any lymph node metastasis exhibits gross extracapsular extension.

c Lymph node staging in AJCC TNM system has remained unchanged in AJCC 6 (2002), AJCC 7 (2010), and AJCC 8 (2017).

Patients were required to have adequate wide excision of the primary. Sentinel lymph node biopsy was required. A complete regional lymphadenectomy was performed if there was any lymph node involvement. Registration within 56 days of the date of surgery was required. Zubrod performance 0–1, adequate renal, hepatic, hematologic, cardiac, and pulmonary function testing were also required. Baseline CT or MRI brain imaging was required and it was suggested that this be repeated every 3 months during protocol participation.

Patients were randomized to receive treatment with either 1 year of high‐dose interferon alpha‐2b (HD‐IFN, Arm 1) or up to three cycles of cisplatin, dacarbazine, vinblastine (CVD), interleukin‐2, and interferon alpha 2b, (BCT, Arm 2) 8. Protocol randomization was stratified by: (1) number of involved nodes; (2) lymph node micrometastases versus macrometastases (including satellite/in‐transit metastases); and (3) ulceration of the primary (yes vs. no). All 402 eligible patients were included in the current analysis. Patient accrual took place between 1 August 2000 and 15 November 2007. Suggested patient imaging included a brain CT or MRI every 3 months. Use of contrast for imaging was not specified in study protocol. Surviving patients were followed up for 10 years.

Data for patients who had disease recurrence were abstracted from case report forms (CRFs) submitted to the SWOG Statistical Center. Permission to access, review, and abstract these data was obtained from the SWOG Melanoma Committee and the SWOG Data Safety Monitoring Committee. CRFs required documentation of metastasis as an indication of progression. Documentation of the specific site of disease progression was optional. However, data concerning the initial site of progression was provided for 176/221 relapsing patients (80%).

Information collected for this analysis included: SWOG patient identification number, date of enrollment, treatment assignment, date off treatment and reason (e.g., completed treatment, toxicity, disease relapse/progression, patient refusal, death, or other specified reason), date of initial progression/relapse, site of initial relapse/progression, date brain metastasis documented (as initial or subsequent site), progression‐free survival, overall survival, survival status, date of death if applicable, institution, and site investigator. Primary tumor characteristics such as depth of invasion, ulceration, nodal, or in‐transit metastases were recorded for all patients as an eligibility requirement. Notations regarding further treatment, additional metastases, and/or cause of death were recorded in a comments section.

### Statistical analysis

A total of 432 patients were initially registered. Twenty‐nine patients were deemed ineligible. In addition, one eligible patient withdrew consent to participate in the study prior to study treatment and was not analyzable for any study endpoints. Thus, data from 402 patients were included in this analysis.

Patients were considered to have relapse/progression in the brain only if the presence of brain metastases and date of onset could clearly be identified on the CRFs. The cumulative incidence of CNS progression in the presence of competing risks of death was estimated and *P*‐values were calculated by fitting a proportional hazards regression model for competing risk distributions using the method of Fine and Gray.

The proportional hazards regression model was also used to evaluate the impact of previously described prognostic factors from studies of locally advanced primary melanoma. These included: an ulcerated primary tumor, compared to no ulceration or unknown ulceration (including unknown primaries); a head and neck primary site, compared to trunk/extremity primaries only, or unknown primary; and presence of any macrometastases (macroscopic lymph nodes, satellite/in‐transit metastases, matted nodal masses) compared to micrometastatic lymph nodes only.

Survival from CNS progression was measured from the documented date of onset of CNS progression to the date of death [9]. Patients last known to be alive were censored at the date of last contact. A Cox proportional hazard model for survival was fit with the number of months from the date of registration to the date of CNS progression [10]. Confidence limits for the medians were constructed using the method of Brookmeyer‐Crowley [11].

## Results

**Figure 1:**
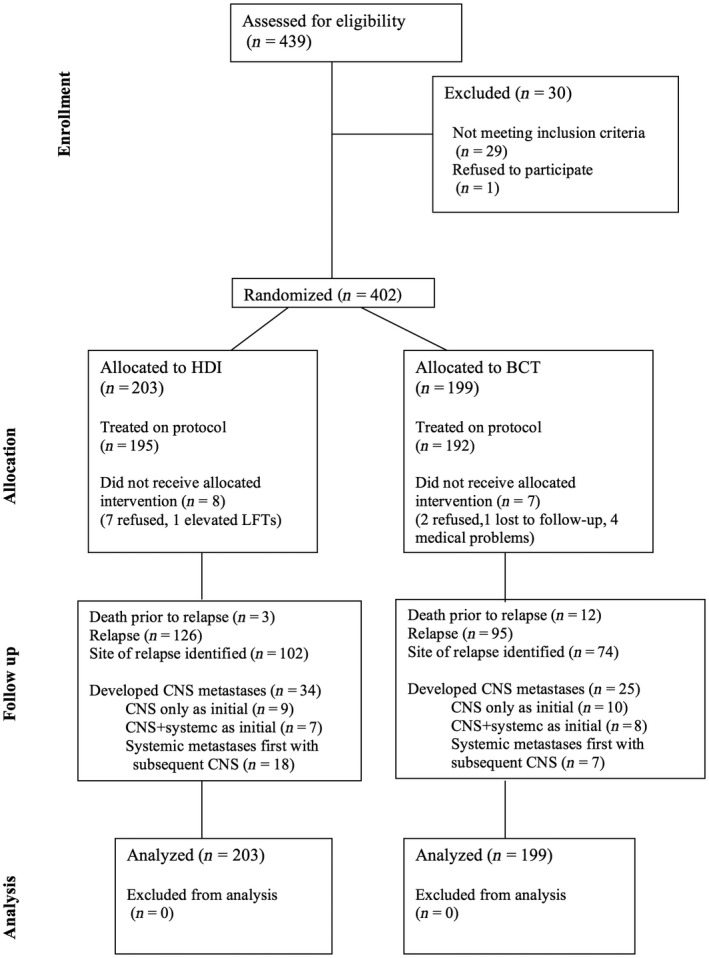
CONSORT flow chart outlining the patient sample used for this retrospective data analysis.

Information for all 402 evaluable patients enrolled on the S0008 clinical trial was screened to identify cases with CNS progression. A CONSORT diagram for patient selection for analysis is shown (Fig. 1). The median follow‐up of patients still alive was over 7 years (range: 1 month to 11 years). The characteristics of patients enrolled on the parent S0008 trial have been published 8.

A total of 221 patients relapsed during the trial (systemic relapse or CNS relapse). This included documented CNS progression occurred in 59/402 of the total number of patients enrolled in this trial (15%), or 27% of patients who relapsed during this trial (59/221). Based on intention to treat analysis, in 34/402 patients (9%) CNS progression represented the initial site of treatment failure. This represents 34/221 of all relapsing patients (15.4%). CNS progression with concurrent systemic progression was a component of initial progression in another 27% of all patients whose melanoma relapsed (59/221). Characteristics of patients with CNS progression are shown (Table 2).

**Table 2:** Characteristics of patients who developed CNS progression

	HD IFN Alfa‐2b (*N *=* *34)		Biochemotherapy (*N *=* *25)	
Gender				
Male	25	74%	20	80%
Female	9	26%	5	20%
Hispanic				
Yes	1	3%	0	0%
No	28	82%	22	88%
Unknown	5	15%	3	12%
Race				
White	33	97%	24	96%
Unknown	1	3%	1	4%
Number of nodes				
1–3 or satellite/in‐transit metastases only	22	65%	18	72%
4+ or any number in combination with satellite/in‐transit metastases	12	35%	7	28%
Nodal involvement type				
Micrometastases only	9	26%	6	24%
Any macrometastases	25	74%	19	76%
Ulceration				
Yes	12	35%	9	36%
No	9	26%	10	40%
Unknown	13	38%	6	24%
Stage				
Stage IIIA (N2a)	3	9%	0	0%
Stage IIIB	14	41%	15	60%
Stage IIIC	17	50%	10	40%

**Figure 2:**
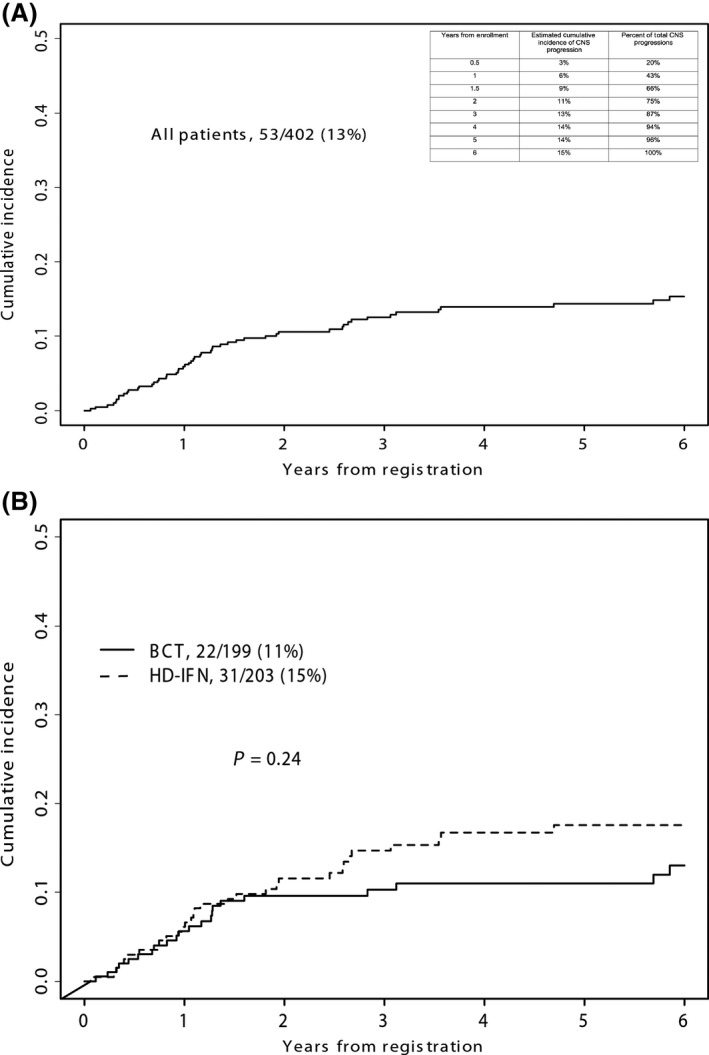
(A) Cumulative incidence of CNS progression in all patients. CNS progression occurred in 59/221 patients who relapsed during the clinical trial (27%). Inset chart shows year‐by‐year incidence. (B) Cumulative incidence of CNS progression by treatment arm. A total of 402 eligible patients were registered to S0008. Brain metastases occurred in 25/199 patients treated with biochemotherapy (BCT) and 34/203 patients treated with high‐dose interferon (HDI).

Among those patients who eventually developed CNS progression, brain metastases developed within 2 years of registration in 73% and within 3 years in 85% (Fig. 2A). All cases of CNS progression occurred within the first 6 years after randomization. The difference in CNS progression was not significant between treatments (25 on BCT, and 34 on HDI, *P *=* *0.24, Fig. 2B). The time to CNS progression was similar in both treatment arms.

**Figure 3:**
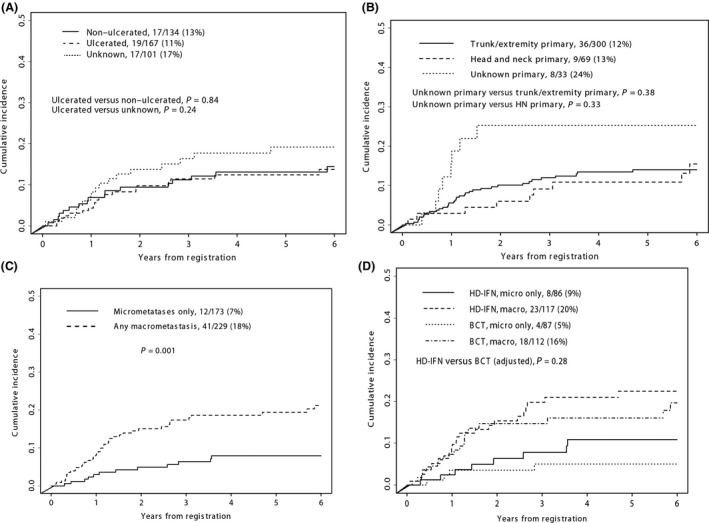
(A) Cumulative incidence of CNS progression related to ulceration of primary tumor. (B) Cumulative incidence of CNS progression related to trunk and extremity, head and neck or unknown primary sites. (C) Cumulative incidence of CNS progression related to macro‐ versus micrometastases. (D) Cumulative incidence of CNS progression by treatment arm, based on macro‐ versus micrometastases.

Ulceration and head and neck primary sites have been proposed as risk factors for development of brain metastases in prior studies [12, 13, 14. In our dataset of 402 patients, there were 167 patients with an ulcerated primary. Five patients had multiple primaries and four of these had at least one ulcerated primary. On exploratory analysis, having an ulcerated primary was not associated with an increased risk of CNS progression when compared to no ulceration (*P *=* *0.84) or unknown ulceration (*P *=* *0.21, Fig. 3A). Sixty‐nine patients had an unknown primary. While there appeared to be a trend toward increased risk for CNS progression in patients with an unknown primary this did not reach statistical significance when compared to trunk or extremity primaries (*P *=* *0.38) or head and neck primaries (*P *=* *0.33, Fig. 3B).

**Figure 4:**
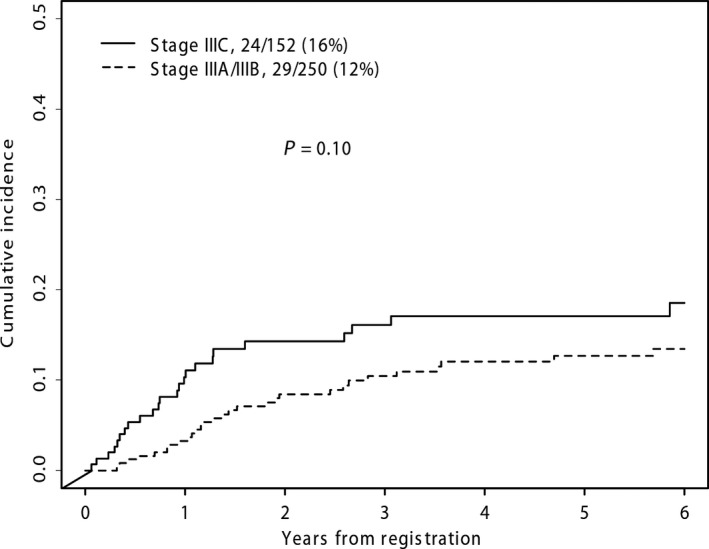
Cumulative incidence of CNS progression by stage. The cumulative incidence of CNS progression in patients enrolled on S0008 was compared between patients with stage stages IIIA(N2a)‐IIIC/disease.

We next evaluated whether presentation with regional macro‐ or micrometastases was associated with an increased risk of CNS progression. This analysis found that the risk of CNS progression was strongly associated with having macroscopic lymph node involvement or in‐transit metastases at presentation, compared to lymph node micrometastases (*P *=* *0.001, Fig. 3C). There was no significant difference in CNS relapse rates between treatments after adjusting for micro‐ versus macrometastases (*P *=* *0.28, Fig. 3D). The cumulative incidence of patients with brain metastases was analyzed by AJCC stage. Stage IIIC patients had an increased rate of CNS progression compared to either Stage IIIA or IIIB patients (*P *=* *0.02, Fig. 4).

**Figure 5:**
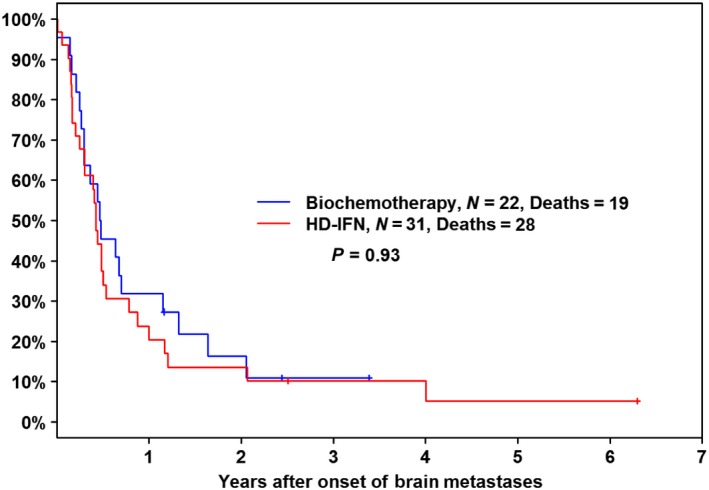
Survival following CNS progression by treatment arm. A Kaplan–Meier plot of survival from the date of diagnosis of CNS progression is shown by treatment arm.

Patients who developed CNS progression had a very high mortality (Fig. 5). In all, 23 of 25 CVD‐BCT patients (92%) and 32 of 34 HDI patients (94%) have died. Median survival after diagnosis of brain metastasis was 6 months (95% confidence interval: 3.2–8.1 months) from the time of onset of brain metastasis in the CVD‐BCT group and 5 months (95% confidence interval: 3.6–6.4 months) in the HDI group. Although we did not expect differences in brain metastasis incidence or outcome between the treatment arms, this possibility was evaluated. Survival following development of brain metastasis was not different between the BCT and HDI treatment arms (*P *=* *0.90).

## Discussion

Currently, at least 10–12% of melanoma patients present with lymph node involvement at the time of initial diagnosis and staging (approximately 7500–9200 patients/year) [15, 16]. Many patients are found to have microscopic involvement limited to 1–3 lymph nodes and arise from a nonulcerated primary (stage IIIA) [17]. We estimated the risk of CNS progression, as defined by brain metastases, in a large cohort of patients with predominantly stage IIIB‐ and IIIC melanoma [18]. Only 5% of the patients enrolled in S0008 were stage IIIA(N2a). Stage IIIA(N1a) patients were ineligible. All patients were systematically staged and underwent uniform surgical management and adjuvant therapy. This mature clinical trial now has a median follow‐up of over 7 years in surviving patients.

Potential risk factors associated with CNS progression have previously been proposed, based on smaller, more heterogeneous studies of patients with primary melanoma. Previously suggested risk factors in early‐stage melanoma included a primary site in the head and neck or an ulcerated primary tumor [7, 12, 13, 14]. In S0008 (Stage IIIAN2a–Stage IIIC patients), neither primary tumor ulceration nor the primary melanoma site were associated with CNS progression. Macrometastatic involvement of lymph nodes or in‐transit metastases, including patients with unknown primaries, proved to be the most important predictors for CNS progression. The importance of macroscopic nodal disease in melanoma patients with an unknown primary as a predictive factor for brain metastases represents a novel observation. There were no apparent differences in the incidence or outcome of brain metastases based on treatment arm. The time to CNS progression also did not differ between arms. This is not unexpected, since neither treatment regimen appeared to have significant CNS activity without addition of radiotherapy [19]. Development of brain metastasis resulted in shortened survival.

A potential limitation of this study is that the parent trial was not designed to prospectively collect data about the site of initial progression. Nonetheless, 80% of CRFs contained this information. There was no information on the initial site of progression in 20% of patients who eventually relapsed and died. This could potentially affect incidence calculations. The CRFs also did not provide information about the percentage of patients who actually underwent recommended CT or MRI scans of the brain every 3 months, or the number who developed symptomatic brain metastases between study visits. It is also not known whether brain imaging was routinely performed at the time of systemic progression. Therefore, the current numbers may underestimate the incidence of CNS relapse. A cautious interpretation is that there is an elevated CNS failure rate in Stage stages IIIAN2a‐IIIC within the first 3 years. Furthermore, our study could guide the design of future prospective studies.

Previously described risk factors for CT detection of synchronous distant metastases after a positive SLN biopsy included primary tumor thickness (*P *=* *0.011), ulceration (*P *=* *0.018), and SLN tumor burden (*P *=* *0.018). Rueth reviewed SEER data from 1992 to 2005. Less than 1/3 of these patients were Stage III melanoma 21. Sequential CT scans in these 1600 patients identified 33% with distant brain metastases over a 5‐year interval. Improvement in survival as a consequence of screening and early detection changed by median of only 2 months in this surgically managed group.

DeRose et al. followed 459 high‐risk melanoma patients. A total of 115 (55%) relapsed either systemically or in the CNS, 101 relapsed prior to 3 years. The median time to recurrence was 12 months (95% confidence interval: 10–16 months) 22. Planned radiographic restaging was performed in 52 patients who reached the 3‐year time point without apparent recurrence was performed. Only two patients with silent systemic relapses were detected at the 3‐year restaging. Both patients became symptomatic within several weeks.

Our current study represents a large prospectively collected clinical dataset consisting of a large number of IIIB and IIIC melanoma patients, who were staged and treated in a uniform fashion. Our data provide a useful benchmark for potential incidence and timing of CNS progression in stage III melanoma. Although a small number of Stage IIIA(N2a) patients were included in S0008, 95% of reported patients had stage IIIB or stage IIIC disease. Since the majority of brain metastases occurred in the first 3 years following surgery, our data suggest the potential usefulness radiographic CNS surveillance during this limited period. Furthermore, our data suggest CNS surveillance in patients with macroscopic nodal disease or in‐transit tumor involvement, who have the greatest risk of CNS progression (approximately 20%). It is likely that current improvements in melanoma therapy will lead to better outcome in treatment of patients with melanoma and early CNS metastases 23.

Screening for an abnormality or disease is only useful if testing is sensitive and if early intervention (prior to symptom development) would make a difference in outcome. It has not yet been convincingly demonstrated that screening and identification of brain lesions would be useful. While sensitive testing modalities (contrast CT or MRI scans) that can identify presymptomatic CNS lesions have become broadly available, historically treatment outcomes for patients with metastatic melanoma with brain metastases were dismal. The historical median survival of patients treated with whole‐brain radiotherapy was only 3–4 months 24, 25, 26. This statistic discouraged most attempts at early detection 21, 22. Fortunately, there have been substantial improvements in ablation of brain metastases and in systemic therapy options for metastatic melanoma that may be capable of influencing survival in patients with CNS relapse 23, 27, 28.

There are signs of progress in treatment of melanoma brain metastases: (1) Most patients diagnosed with melanoma brain metastases now have only 1–3 metastases, rather than dozens of brain lesions at one time 29, 30. (2) Improvements in radiosurgery incorporating computerized imaging and targeting, which allows precise, high‐dose‐rate treatment of metastases, sparing normal tissues 31. (3) Treatment outcomes in patients with melanoma brain metastases patients are improving. A median survival exceeding 9 months has been reported for patient with ≤3 metastases and 5.7 months for >3 metastases 32. (4) Treatment of smaller, asymptomatic lesions (e.g., <3–5 mL) detected during surveillance produces a high radiosurgery local control rate, while larger, symptomatic, lesions appear less responsive 33, 34, 35, 36, 37. (5) There are new systemic therapy options that have markedly improved survival for patients with metastatic melanoma 38, 39, 40, 41, 42, including significant responses in patients with brain metastases 28, 43, 44. These recent advances raise the possibility that screening and treatment of melanoma brain metastases perhaps should be revisited in a well‐designed clinical trial in high‐risk Stage IIIB and IIIC to test whether early detection and treatment of brain metastases can produce improved treatment outcomes, including quality of life and survival.

## Conflict of Interest

None of the authors reported a direct conflict of interest related to the manuscript topic.
